# Characterization of the complete chloroplast genome of *Amomum longiligulare* (Zingiberaceae)

**DOI:** 10.1080/23802359.2019.1637295

**Published:** 2019-07-12

**Authors:** Zhong-Ji Li, Jie Zhang, Ying-Ying Liu, Xiao-Li Liu, Guo-Dong Li, Zi-Gang Qian

**Affiliations:** a Faculty of Traditional Chinese Pharmacy, Yunnan University of Chinese Medicine, Kunming, China;; c Yunnan Key Laboratory for Dai and Yi Medicines, Yunnan University of Chinese Medicine, Kunming, Yunnan, China;; b Yunnan Insitute for Food and Drug, Kunming, China

**Keywords:** *Amomum longiligulare*, complete chloroplast genome, phylogenetic analysis

## Abstract

*Amomum longiligulare* T. L. Wu (Zingiberaceae) is a herbaceous perennial grown in Hainan Province, China, which is an important medicinal plant used for the improvement of gastrointestinal motility. The complete chloroplast genome of *A. longiligulare* was assembled based on next-generation sequencing. The plastome was a quadripartite circular with 16,3608 bp in length, including two inverted repeat (IR, 22,696 bp) regions, one large single-copy region (LSC) and one small single-copy region (SSC) of 88,680 bp and 29,536 bp, respectively. The chloroplast genome contained 123 genes, including 85 protein-coding genes, 30 tRNA genes, and 8 rRNA genes. The overall GC content of the whole genome is 36.1%. Phylogenetic analysis strongly supported *A. longiligulare* and its congeneric species, *A. kravanh* and *A. compactum*, as sister group with 100% bootstrap value.

*Amomi fructus*, known as Sharen, is an important Traditional Chinese Medicine with thousands of years of medical use history to relieve a range of gastrointestinal diseases in China (Commission of Chinese Pharmacopoeia 2015). The dried ripe fruits of *Amomum villosum* Lour., *A. villosum* Lour. var. xanthioides T. L., and *A. longiligulare* T. L. Wu were principal resources of Sharen. Among those three species, *A. longiligulare* is a herbaceous perennial endemic to Hainan Province, China. Due to its extremely low natural fertility as well as the increasing wild harvesting to meet the growing demand of the market, the natural populations of *A. longiligulare* decreased rapidly in recent years (Li and Wu 2012). Facing the shortage of wild *A. longiligulare*, artificial cultivating and natural fostering have been practiced in recent years. Till now, the genomic information for *A. longiligulare* is extremely limited. Here, we sequenced the cp genome for *A. longiligulare* and analyzed the genome features. This information will be useful for the conservation and breeding program.

Fresh leaves of *A. longiligulare* were collected from Hainan Branch Institute of Medicinal Plant Development, Chinese Academy of Medical Sciences (18°44'N, 110°12'E), and voucher specimens (HN003) were deposited in the Herbarium of Yunnan University of Chinese Medicine. A sequence library was constructed and sequencing was performed using the Illumina HiSeq 2500-PE150 platform (Illumina, San Diego, CA, USA). All raw reads were filtered by using NGS QC Toolkit_v2.3.3 with default parameters to obtain clean reads (Patel and Jain 2012). The plastome was *de novo* assembled using NOVOPlasty (Dierckxsens et al. 2017). The complete cp genome was annotated with the online annotation tool GeSeq (Tillich et al. 2017). All of the plastomes were aligned using MAFFT v.7 (Katoh and Standley 2013), and the RAxML (Stamatakis 2014) inference was performed using GTR model with support for branches evaluated by 1000 bootstrap replicates. The phylogenetic analysis was conducted based on 10 published chloroplast genomes to infer phylogenetic position of *A. longiligulare* within the family of Zingiberaceae.

The complete chloroplast genome of *A. longiligulare* was 163,608 bp in length (GenBank accession number MK 889505), and includes a large single-copy (LSC) region of 88,680 bp and a small single-copy (SSC) region of 29,536 bp, separated by two inverted repeat (IR) regions of 22,696 bp, respectively. And, it contained 123 genes, including 85 protein-coding genes, 8 rRNA genes, and 30 tRNA genes. The overall GC content of 36.1%. A total of 74 SSRs were discovered by the online software IMEx (Mudunuri and Nagarajaram 2007). Among them, the numbers of mono-, di-, tri-, tetra-, penta- and hexa- nucleotides SSRs are 33, 16, 6, 14, 3, and 2, respectively.

The phylogenetic tree showed that species from the Zingiberaceae formed a monophyletic clade (Figure 1). The result strongly supported *A. longiligulare* and its congeneric species, *A. kravanh*, and *A. compactum*, as sister group with 100% bootstrap value. The data will provide a useful resource for studying the genetic diversity of *A. longiligulare*, the phylogenetic relationships of the Zingiberaceae family, and conservation of this valuable species.

**Figure 1.**
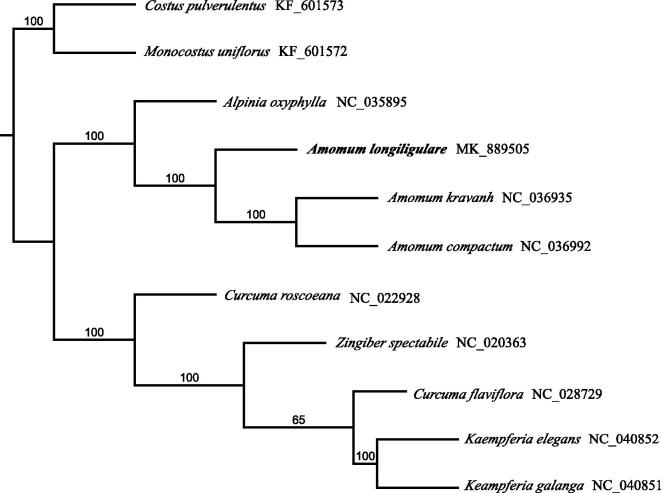
The plastome phylogeny of Zingiberaceae. Bootstraps were shown next to the node.
